# NF-κB-mediated astrocyte dysfunction initiates neurodegeneration

**DOI:** 10.18632/oncotarget.18320

**Published:** 2017-05-31

**Authors:** Michael Lattke, Stephanie N. Reichel, Bernd Baumann

**Affiliations:** Institute of Physiological Chemistry, University of Ulm, Ulm, Germany

**Keywords:** NF-κB, Bergmann glia, neuroinflammation, neurodegenerative disease

The IKK/NF-κB signal transduction and transcription factor system is a central regulator of inflammation, cell survival and differentiation, and is implicated in multiple human pathologies including multiple sclerosis, glioblastoma and neurodegenerative disorders. The IKK/NF-κB system is prominently activated by proinflammatory cytokines and molecules derived from pathogens or damaged cells and NF-κB target genes like cytokines and chemokines drive inflammatory responses aimed to restore cell and tissue homeostasis.

Astrocytes, the most abundant glial cell type in the brain, have important functions for the homeostasis and survival of neurons. In addition, astrocyte-related cell types act as neural stem cells, and in case of ependymal cells facilitate the flow of the cerebrospinal fluid through the cerebral ventricles with motile cilia. Importantly, astrocytes respond strongly to brain injury and other pathological conditions, exerting important inflammatory and immune-related functions to restore brain homeostasis. However, these responses can also disrupt their physiological functions or promote excessive neuroinflammation [1]. Therefore, inflammation-associated dysfunction of astrocytes and astrocyte-related cells has been proposed to contribute to the pathogenesis of a large spectrum of neurological disorders, e.g. multiple sclerosis, amyotrophic lateral sclerosis, spinocerebellar ataxias or hydrocephalus.

In line with its crucial role in proinflammatory responses, genetic inactivation of NF-κB signaling in astrocytes was found to ameliorate neuroinflammation in mouse models of spinal cord injury and multiple sclerosis, which resulted in reduced neurological deficits [2, 3]. However, whether astroglial IKK/NF-κB signaling is a key driver or only a modulator of neuroinflammation and pathogenesis in these specific conditions remains unclear. Furthermore, the role of NF-κB in astrocytes in brain development and homeostasis in normal physiological conditions is not well understood.

Therefore, we developed a conditional transgenic mouse model allowing the doxycycline-regulated expression of a constitutively active allele of the key NF-κB inducing kinase IKK2 specifically in astrocytes. Remarkably, IKK2 activation in astrocytes during brain development is sufficient to induce neuroinflammation through the upregulation of a large set of proinflammatory cytokines, chemokines, cell adhesion molecules and acute phase response factors, demonstrating a crucial role of astroglial NF-κB signaling as a driver of neuroinflammation [4]. This phenotype was accompanied by defects in brain development, most strikingly prominent hydrocephalus formation, a pathology frequently associated with neuroinflammatory conditions. Hydrocephalus was associated with an impaired maturation of the astrocyte-related ependymal cells [4], which failed to develop motile cilia, a defect shown to cause hydrocephalus in other models.

To address the function of astroglial NF-κB signaling in the adult brain, we activated IKK2 after completion of brain development to avoid such developmental defects. In this paradigm, global neuroinflammation arises in the adult animal, which surprisingly did not result in acute neurological abnormalities, nor did it aggravate neurodegeneration in a model of Parkinson’s disease [5]. However, with some delay the animals with active IKK2 in astrocytes developed motor deficits, linked to a severe loss of Purkinje neurons in the cerebellum, a main center of motor coordination [6]. Interestingly, such a phenotype is observed in a group of neurodegenerative disorders (cerebellar ataxias), which include genetic disorders (e.g. spinocerebellar ataxias) and autoimmune/inflammatory disorders affecting the cerebellum, like the cancer-associated paraneoplastic disorders and multiple sclerosis [7]. Although T-cell mediated mechanisms have been proposed to drive Purkinje cell degeneration in these inflammatory disorders, the pathogenic mechanisms are not well understood. Our study now indicates that IKK2/NF-κB activation in astrocytes, which occurs in diverse inflammatory CNS conditions, might be a critical step in the pathogenesis of inflammatory cerebellar ataxias [6].

**Figure 1:**
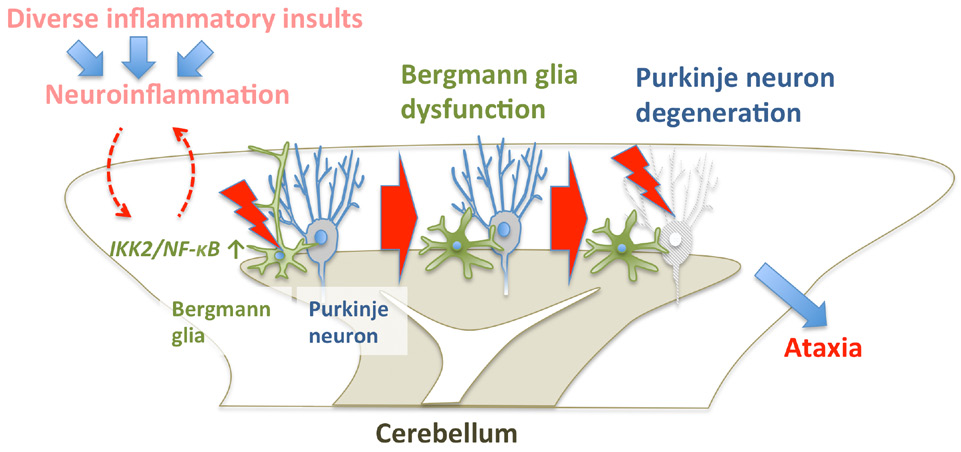
IKK2/NF-κB-mediated Bergmann glia dysfunction causes non-cell-autonomous Purkinje neuron degeneration and ataxia

We could show that IKK2/NF-κB activation in Bergmann glia, a specific radial glia-like population of cerebellar astrocytes, irreversibly disrupts their local interaction with Purkinje cells, finally resulting in Purkinje cell degeneration (Figure 1). Defects in this specific neuro-glial interaction also cause Purkinje cell death in other genetic mouse models and might explain the selective vulnerability of Purkinje cells to degeneration in cerebellar ataxias. The identification of effector mechanisms of IKK2/NF-κB activation responsible for disruption of Bergmann glia function will therefore be important to develop specific therapies for these disorders. Interestingly, we found that IKK2 activation disrupts the specialized morphology of Bergmann glia and causes downregulation of the glial glutamate transporters EAAT1 and EAAT2 [6], which is thought to cause extracellular accumulation of the neurotransmitter glutamate, thus promoting neurodegeneration by excitotoxicity.

Such a disruption of mature astrocyte morphology and function, along with a gain of innate immune cell-like functions and in some conditions cell cycle re-entry/proliferation, which occurs in many neuroinflammation-associated disorders, is called astrogliosis and is suggested to resemble a partial de-differentiation of astrocytes. Together with our earlier finding that NF-κB activation prevents fully functional differentiation of astrocyte-related ependymal cells, the question arises, whether NF-κB activation more broadly counteracts maturation of astrocyte-related cells. Indeed, a recent study supports this idea [8], and many other studies show that NF-κB signaling promotes progression of glioblastoma, a type of cancer believed to originate from de-differentiated astrocytes or related neural stem/progenitor cells.

Beside the role of astroglial IKK2/NF-κB signaling in brain development and homeostasis described here, our conditional mouse model will be a valuable tool to address the role of neuroinflammation and astroglial NF-κB signaling in neurodegenerative and autoimmune disorders as well as brain cancers. Finally, the conditional regulation of NF-κB activation in astrocytes will be instrumental to dissect their role in the crosstalk with other immunocompetent cells like microglia in more detail.
